# Effects of Stair-Climbing Exercise Snacks with Blood Flow Restriction on Fitness in Sedentary University Students: A Pilot Randomized Controlled Trial

**DOI:** 10.3390/life16081226

**Published:** 2026-07-24

**Authors:** Longzhen Qiu, Jiling Liang, Ling Yu, Siyao Gao, Yineng Tan, Xueliang Li, Jianyu Gan

**Affiliations:** 1 Department of Physical Education, Central South University, Changsha 410083, China; qiulongzhen2016@gmail.com (L.Q.); jilingliang@csu.edu.cn (J.L.); gaosiyao@csu.edu.cn (S.G.); 2 School of Strength and Conditioning Training, Beijing Sport University, Beijing 100084, China; 2023210116@bsu.edu.cn; 3 Sports Coaching College, Beijing Sport University, Beijing 100084, China; 2019010250@bsu.edu.cn

**Keywords:** exercise snacks, stair climbing, blood flow restriction training, sedentary behavior, physical fitness

## Abstract

Time-efficient strategies are needed to enhance physical fitness in sedentary university students. This pilot study examined the preliminary effects of maximal-effort stair-climbing exercise snacks performed alone (ESA) or combined with practical blood flow restriction (ES + BFR). Twenty-three sedentary male university students were included in the per-protocol analysis (ESA, *n* = 7; ES + BFR, *n* = 8; control, *n* = 8). Over 4 weeks, both intervention groups completed 48 supervised sessions, each consisting of three 20 s maximal stair climbs separated by 2 min rest. Assessed outcomes were forced vital capacity (FVC), 50 m sprint time, 1000 m run time, standing long jump (SLJ), sit-and-reach flexibility, and body mass index (BMI). Group × Time interactions were tested using mixed-model ANOVA. Significant interactions emerged for all metrics (*p* ≤ 0.01, partial η^2^ = 0.35–0.70), except BMI. Both interventions significantly improved FVC, flexibility, 1000 m run and 50 m sprint performance (*p* < 0.05; absolute Cohen’s *d* range = 1.01–2.68). Although no significant between-group differences were observed, ES + BFR produced larger within-group effect sizes than ESA for 1000 m run time (*d* = −2.14 vs. −1.66) and 50 m sprint time (*d* = −2.21 vs. −1.89). Only ES + BFR significantly increased SLJ (*p* < 0.05), whereas the control group exhibited a significant decline (*p* < 0.05). BMI remained unchanged. The 4-week stair-climbing exercise snack protocol is feasible and may improve pulmonary function, endurance, speed, and flexibility in sedentary male students. Adding practical BFR shows promising numerical benefits for sprint, power, and endurance performance, but these exploratory findings require confirmation in larger trials.

## 1. Introduction

Sedentary behavior has become a hallmark of contemporary life, and university students are disproportionately affected. Meta-analytic evidence indicates that they spend 7.29 h per day in sedentary activities, which is higher than in the general young adult population, and that this time has risen over the past decade [1]. Given this high volume and its rising trajectory over the past decade, the consequent erosion of physical fitness in this population is concerning. Empirical evidence shows that sedentary or low-activity behavioral patterns are significantly associated with declines in health-related physical fitness, while a higher body mass index (BMI) is linked to poorer strength, coordination, and overall fitness [2]. Furthermore, as physical activity is inversely related to total sitting time in this demographic [3], promoting physical activity to interrupt prolonged sitting constitutes a primary target for future interventions. Moreover, beyond the direct effects on physical fitness, sedentary lifestyles are strongly associated with elevated risks of cardiovascular disease, metabolic disorders, and all-cause mortality [4] and are linked to impaired mental health and cognitive function, including higher levels of anxiety and depression, as well as impaired self-regulation [5]. Recent evidence indicates that sleep quality, potentially compromised by sedentary behavior, can impede cognitive performance in university students [6]. Although even moderate activity can mitigate these risks [7], only a minority of university students meet the guideline of 150 min of moderate-to-vigorous physical activity per week [8]. Time constraints, low motivation, and limited access to exercise facilities are the main barriers to regular physical activity [9], highlighting the need for practical, time-efficient, and accessible exercise solutions tailored to this population.

In response, interest has grown in time-efficient strategies such as exercise snacks. These constitute a subtype of short bouts of accumulated exercise (SBAE), broadly defined as any physical activity of any intensity and mode, with each bout lasting ≤10 min and performed multiple times daily with adequate recovery intervals [10]. Exercise snacks specifically refer to isolated bouts of vigorous exercise lasting ≤1 min and performed periodically throughout the day [10]. Evidence indicates that regular exercise snacks could improve cardiorespiratory fitness [10,11], muscular strength [10] and peak power output [11] while counteracting the metabolic harms of prolonged sedentary behavior [10,11,12]. Moreover, the exercise-snacks format uses short, frequent bouts to provide sustained physiological stimulation without excessive fatigue [13] and has also been shown to improve mood, reduce perceived fatigue, and enhance cognitive performance [14], thereby reducing time and motivation barriers and improving adherence in inactive populations. Stair-climbing sprinting, a common form of exercise snacks, has been shown to improve cardiorespiratory fitness [15,16] and reduce body fat [17] in sedentary populations; however, evidence for its effects on other physical performance metrics, such as lower-limb explosive power and sprinting ability, remains limited.

Blood flow restriction (BFR) training is another practical and time-efficient strategy that may enhance training adaptations under low-load conditions. This modality involves applying a tourniquet or inflatable cuff to partially restrict blood flow to the working skeletal muscles during exercise. By limiting circulation, BFR accelerates metabolite accumulation and creates a localized hypoxic environment, which may be beneficial in high-load training with long rest intervals [18]. This stimulus has been proposed to increase metabolic stress and local hypoxic signaling, which may support muscular and endurance-related adaptations under low-load conditions. For instance, BFR performed at intensities as low as 20% of one-repetition maximum has been shown to effectively enhance muscle function and aerobic capacity [19]. Compared with traditional high-intensity training, BFR has been suggested to improve muscle strength and endurance within shorter time frames, offering the potential advantages of low load and high efficiency [20]. Accumulating evidence supports the substantial research potential and practical applicability of BFR across diverse populations [21,22]. However, practical methods such as the use of elastic wraps tightened by perceived tightness introduce stimulus variability [23], and safety concerns (e.g., contraindications in hypertension or thromboembolism risk) require careful participant screening and supervision [24].

The combination of stair-climbing exercise snacks and blood flow restriction presents a particularly promising yet relatively unexplored strategy. Stair climbing inherently engages high power output and activates large muscle groups, while BFR can amplify the local metabolic stress and hypoxic signaling within those muscles, even during brief exertions. However, the short-term efficacy of stair-climbing exercise snacks in sedentary young adults is not well established, and it is unclear whether combining exercise snacks with BFR yields additive or synergistic benefits. We reasoned that this combined stimulus could be uniquely effective for simultaneously improving aerobic endurance, sprint speed, and lower-limb explosive power, which are fitness components that typically require longer or separate training modalities. To test this, the present pilot study examined the initial effects of a four-week stair-climbing exercise-snack protocol performed alone (ESA) or in combination with practical blood flow restriction (ES + BFR) on physical fitness outcomes in sedentary male university students. We hypothesized that both ESA and ES + BFR would improve key fitness parameters and that ES + BFR would exhibit more pronounced effects, particularly for lower-limb power, sprint performance, and aerobic endurance.

## 2. Materials and Methods

### 2.1. Study Design

**Figure 1:**
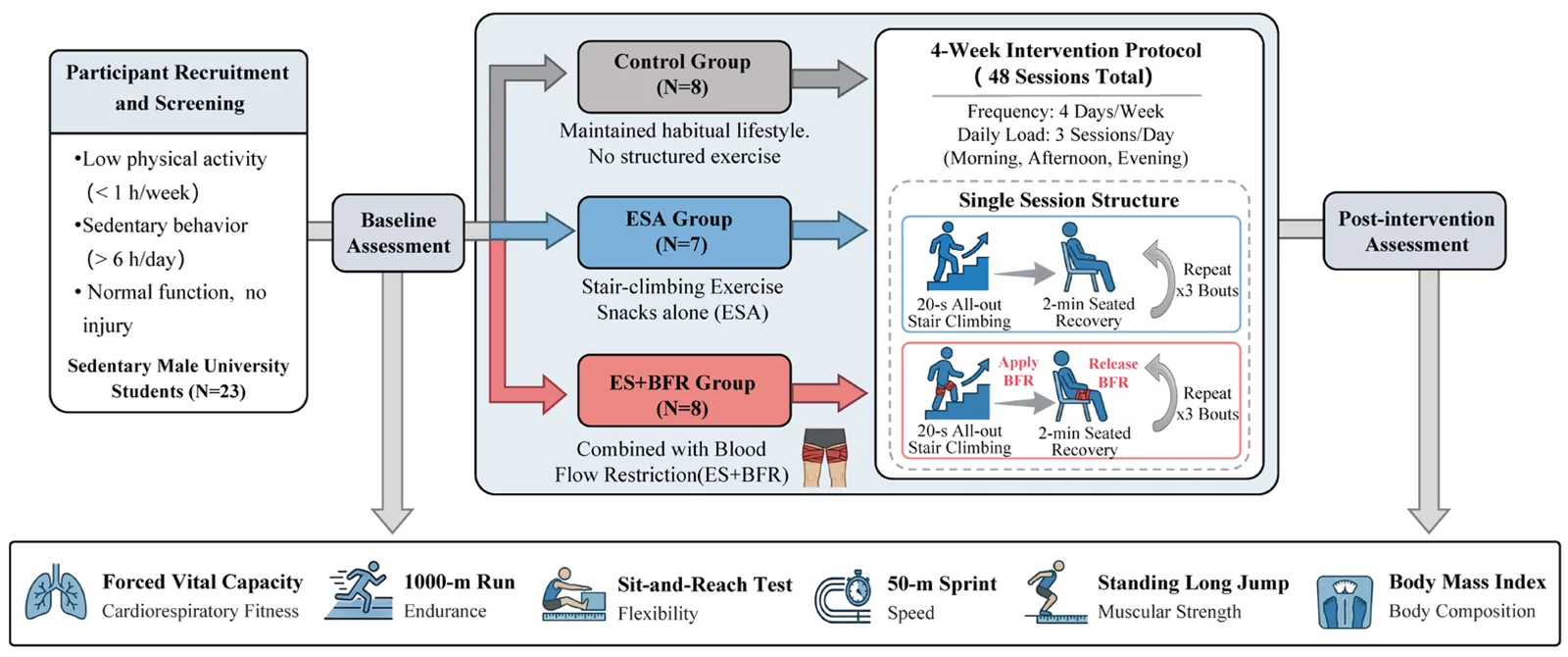
Flowchart of the study design and grouping.

This pilot study employed a three-arm, parallel-group, randomized controlled trial (RCT) design. A flowchart depicting the study design and grouping is shown in Figure 1. Participants were randomly allocated to one of three groups using a computer-generated sequence: stair-climbing exercise snacks alone (ESA), stair-climbing exercise combined with blood flow restriction (ES + BFR), or a control group. Allocation was concealed via sequentially numbered, opaque, sealed envelopes prepared by an independent researcher, and the principal investigator opened them only after all baseline assessments were completed. The 4-week intervention included pre- and post-intervention assessments conducted under standardized conditions at the same time and location. The attendance and completion of all training sessions were recorded by the same researcher. The study protocol was approved by the Ethics Committee of Central South University of China (Approval No. 2023071203) and conducted in accordance with the Declaration of Helsinki. Written informed consent was obtained from all participants prior to enrollment. As this was a pilot study designed to primarily assess feasibility and gather exploratory effect-size estimates rather than to provide a definitive test of efficacy, the trial was not registered.

### 2.2. Sample Size and Participants

Data collection spanned March–May 2025, with a continuous 28-day intervention period for each participant. A priori sample-size calculation was performed using G*Power (Version 3.1.97). For a three-group, repeated-measures design (effect size (f) = 0.25, α error = 0.05, and power = 0.80), a minimum of 18 participants per group was required. However, due to the intensive nature of the supervised 4-week intervention (48 sessions) and the logistical constraints of the academic semester, recruitment fell short of this target. This calculation was used as a reference for a fully powered efficacy trial. Because the present study was conducted as a pilot trial and the target sample was not reached, all efficacy-related findings were interpreted as exploratory rather than confirmatory.

Eligibility criteria included: (1) participation in exercise or sport once or less per week [25]; (2) daily sedentary behavior >6 h or ≥2 consecutive hours of uninterrupted sitting [26]; (3) normal physical and cognitive function; and (4) no history of musculoskeletal injury, chronic disease, or medication use known to affect exercise capacity.

A total of 32 male university students were screened for eligibility. Five participants were excluded before randomization because they did not meet the eligibility criteria or were unable to participate in the full intervention schedule. Consequently, 27 eligible participants were randomized to the ES + BFR, ESA, or control group.

**Figure 2:**
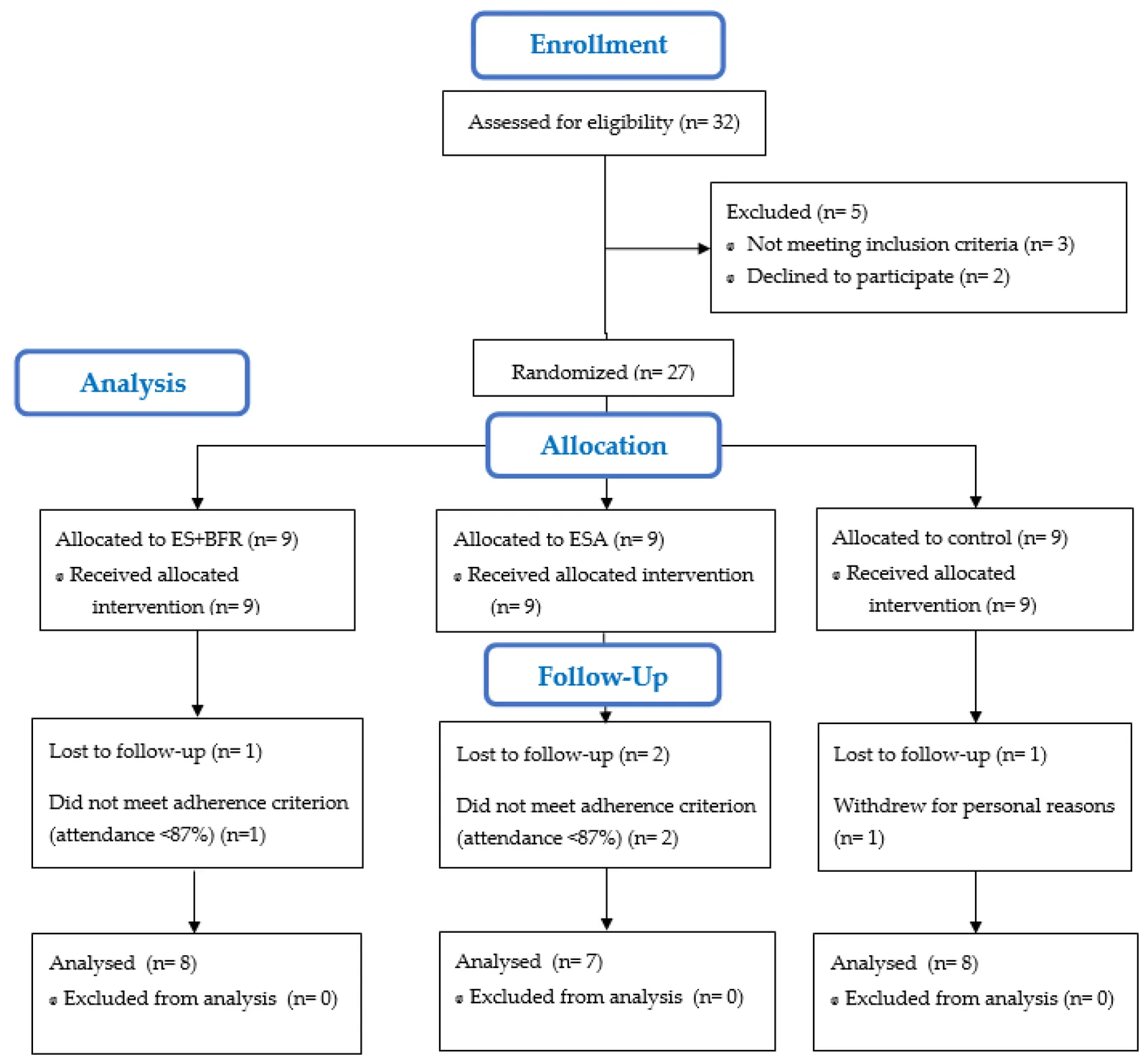
CONSORT 2025 flow diagram.

During the intervention period, two participants in the control group withdrew for personal reasons, and three participants in the intervention group failed to meet the prespecified adherence criterion. Adherence was defined a priori as attendance at ≥87% of supervised sessions, corresponding to at least 42 of the 48 planned sessions [27]. This cutoff is consistent with systematic review evidence that ~80–90% completion is a standard per-protocol criterion to ensure a minimum effective dose in supervised exercise trials [27]. Therefore, 23 participants were included in the per-protocol analysis: ES + BFR (*n* = 8), ESA (*n* = 7), and control (*n* = 8) (as shown in Figure 2).

Given the pilot design, progression criteria for a future trial (e.g., recruitment, retention, adherence, safety, and effect size) were not pre-specified; instead, we derived post hoc benchmarks from the current data. Because the final per-protocol sample was smaller than the target, the findings should be interpreted as early hypothesis-generating evidence. The results are primarily intended to inform feasibility, variance estimates, effect-size estimation, and sample-size planning for future fully powered trials.

### 2.3. Assessments

To capture fitness components that are commonly compromised by a sedentary lifestyle and are linked to health risk, we assessed the following outcomes. Four co-primary outcomes were designated across distinct fitness domains: (1) forced vital capacity (FVC; pulmonary function), (2) 1000 m run time (aerobic endurance), (3) 50 m sprint time (speed), and (4) standing long jump (SLJ; lower-limb explosive power). Secondary outcomes were sit-and-reach flexibility and body mass index (BMI). FVC is an indicator of pulmonary function associated with all-cause mortality [28] and was measured using a spirometer (HK6800B-FH, Hengkang Jiaye, Shenzhen, China). The 1000 m run, a validated field test from the Chinese National Student Physical Health Standard [29], was performed on a 400 m outdoor track with instructions to finish as quickly as possible. The 50 m sprint was timed with dual-beam electronic gates (G4 Timing Gates, SWIFT Performance, Wacol, QLD, Australia); participants completed two maximal sprints from a standing start with at least 3 min recovery, and the faster time was retained. SLJ distance was measured from a fixed take-off line to the nearest heel contact, with the best of three attempts recorded to 0.1 cm. Posterior-chain flexibility was assessed via the sit-and-reach 3test (HK6800-TQ, Hengkang Jiaye, Shenzhen, China), retaining the best of three forward reaches; reduced flexibility in sedentary individuals is linked to functional limitations and low back pain [30]. BMI was calculated as weight divided by height squared (kg/m^2^) and recorded as a basic anthropometric descriptor. All fitness outcomes were evaluated at baseline and within 48 h after the intervention. Participants were instructed to maintain habitual diet, sleep, and daily routines and to refrain from vigorous exercise, alcohol, and caffeine for 24 h before testing. Compliance was monitored via weekly self-reports; no major deviations were reported.

### 2.4. Training Protocol

Participants in both intervention groups completed a stair-climbing exercise-snack protocol, comprising 3 × 20 s all-out stair-climbing bouts per session interspersed with 2 min of seated recovery. Stair climbing was performed on an indoor staircase under supervision. The staircase consisted of 22 steps per flight, with each step measuring approximately 30 cm in width and 15 cm in depth. Participants were instructed to climb as many steps as possible within each 20 s bout while maintaining safety. Handrail use was permitted when necessary. Participants wore their usual athletic shoes and sportswear. Before baseline testing and the first training session, all participants completed a familiarization session to practice the stair-climbing procedure and testing protocols. Sessions were conducted three times daily (morning, afternoon, and evening), four days per week, totaling 48 sessions over four weeks. Before each training session, participants completed a standardized 3 min warm-up consisting of light walking and dynamic lower-limb movements. All sessions were directly supervised by the same researcher. Session completion was recorded using attendance logs, and the number of completed stair steps or flights in each 20 s bout was recorded. Standardized verbal instructions were provided before each bout, and no additional motivational feedback was given beyond pre-defined safety and effort cues. Participants in the control group were instructed to maintain their usual lifestyle and refrain from starting any new structured exercise program during the 4-week study period.

For the ES + BFR group, blood flow restriction was applied using non-pneumatic elastic wraps (5 cm width) secured around the proximal thighs. During each exercise bout, participants manually tightened the wraps to a perceived tightness of approximately 7 on a 0–10 scale, where 0 indicated no pressure and 10 indicated maximal tolerable pressure; wraps were loosened during rest intervals. Before every 20 s climb, the researcher confirmed the target tightness and verified the absence of pain, numbness, and excessive discomfort [31]. Verbal instructions and familiarization were standardized to promote within-protocol consistency. Comfort, skin condition, and adverse events were monitored throughout each supervised session [23]. Given the pilot nature of this trial, which aimed to assess the feasibility and initial signals of a combined exercise snack-plus-practical BFR regimen, this perceptually regulated approach was adopted as a field-appropriate alternative to Doppler ultrasound-based arterial occlusion pressure.

### 2.5. Statistical Analysis

The data are expressed as group means ± standard deviations (SDs). Distributional normality was assessed within each group using the Shapiro–Wilk test, and homogeneity of variance was examined with Levene’s test. Training effects were evaluated via two-way mixed-model repeated-measures ANOVA with factors of Group (ES + BFR, ESA, and Control) and Time (pre and post), and the main effects of Group and Time, as well as the Group × Time interaction, were tested. Partial eta-squared (η^2^p) is reported as an effect-size measure for all significant interaction effects. When omnibus effects were significant, Bonferroni-adjusted pairwise comparisons (simple effects on estimated marginal means) were performed.

For statistically significant within-group pre–post changes, Cohen’s d effect sizes with 95% confidence intervals were calculated and interpreted as trivial (*d* < 0.20), small (0.20 ≤ *d* < 0.60), moderate (0.60 ≤ *d* < 1.20), large (1.20 ≤ *d* < 2.00), very large (2.00 ≤ *d* < 4.00), or extremely large (*d* ≥ 4.00) [32]. Effect sizes are not reported for non-significant pre–post changes. Due to the small sample size (*n* = 23), effect-size estimates should be interpreted cautiously, as they may be inflated and are primarily intended to inform sample-size planning for future trials rather than to provide definitive estimates of true effects [33]. All analyses were performed on the per-protocol sample. Because this pilot trial did not use intention-to-treat analysis, the potential for attrition bias was considered when interpreting the findings. Statistical significance was set at *p* < 0.05. All analyses were conducted in SPSS version 24 (IBM, Armonk, NY, USA).

## 3. Results

Baseline characteristics and attendance are summarized in Table 1. Twenty-three participants were included in the per-protocol analysis. Baseline characteristics were comparable among the three groups (Table 1). One-way ANOVA confirmed no significant between-group differences at baseline for any variable (all *p* > 0.05). Session attendance was exceptionally high in both intervention groups, with mean attendance of 47.63 ± 0.74 sessions (99.2%) in ES + BFR and 47.29 ± 1.25 sessions (98.5%) in ESA, indicating excellent feasibility of the supervised protocol.

### 3.1. ANOVA Results

Table 2 reports the omnibus ANOVA results (main effects of Group, Time, and Group × Time interactions). Significant main effects of Time were observed for FVC, 1000 m run time, 50 m sprint time, and sit-and-reach distance (all *p* < 0.05). There were no significant main effects of Group for any variable (all *p* > 0.05). Notably, significant Group × Time interactions were observed for FVC, 1000 m run time, 50 m sprint time, sit-and-reach flexibility, and SLJ (*p* ≤ 0.01, partial η^2^ = 0.35–0.70).

### 3.2. Post Hoc Within-Group Comparisons

Table 3 presents pre- and post-intervention means, standard deviations, and within-group Cohen’s d effect sizes with 95% confidence intervals.

#### 3.2.1. Forced Vital Capacity

**Figure 3:**
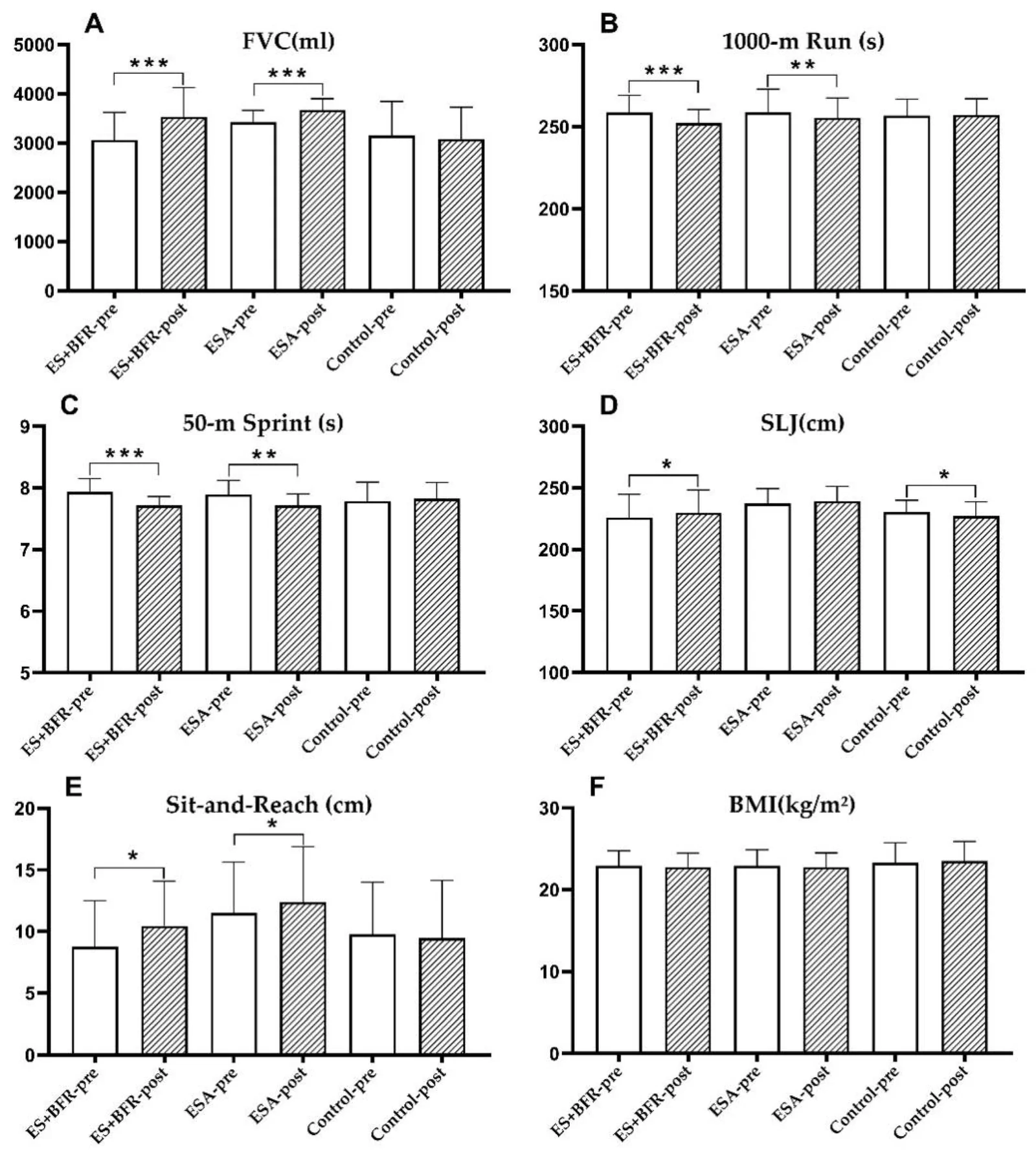
Pre- and post-intervention comparisons of fitness indicators among ES + BFR, ESA, and control groups. (**A**) Forced vital capacity (FVC, ml); (**B**) 1000-m Run (s); (**C**) 50-m sprint (s); (**D**) Standing long jump (SLJ, cm); (**E**) Sit-and-Reach (cm); (**F**) Body mass index (BMI, kg/m^2^). * *p* < 0.05, ** *p* < 0.01, and *** *p* < 0.001 vs. baseline within the same group. Data are presented as mean ± SD. ES + BFR, exercise snacks with blood flow restriction; ESA, exercise snacks alone; FVC, forced vital capacity; SLJ, standing long jump; BMI, body mass index.

As shown in Table 3 and Figure 3, FVC increased significantly in both ES + BFR (+474.5 mL, *p* < 0.001, *d* = 2.48, 95% CI [1.08, 3.88]) and ESA (+253.4 mL, *p* < 0.001, *d* = 2.68, 95% CI [1.09, 4.26]). The control group showed no significant change (*p* = 0.24). No significant between-group difference was observed between ES + BFR and ESA (*p* = 0.18).

#### 3.2.2. 1000 m Run

The post-intervention reductions are summarized in Table 3 and visually displayed in Figure 3. Run time decreased significantly (improved) in both ES + BFR (−6.25 s, *p* < 0.001, *d* = −2.14, 95% CI [−3.40, −0.88]) and ESA (−3.44 s, *p* = 0.009, *d* = −1.66, 95% CI [−2.80, −0.52]). The control group showed no significant change (*p* = 0.68). Although ES + BFR demonstrated a larger numerical effect size than ESA (*d* = −2.14, very large vs. *d* = −1.66, large), the between-group difference was not statistically significant (*p* = 0.42).

#### 3.2.3. 50 m Sprint

Table 3 and Figure 3 present the pre-to-post changes in 50 m sprint performance. Sprint time decreased significantly (improved) in both ES + BFR (−0.23 s, *p* < 0.001, *d* = −2.21, 95% CI [−3.50, −0.92]) and ESA (−0.18 s, *p* = 0.003, *d* = −1.89, 95% CI [−3.43, −0.32]). The control group showed no significant change (*p* = 0.55). Although ES + BFR showed larger numerical effect sizes than ESA (*d* = −2.21, very large vs. *d* = −1.89, large), the between-group difference was not statistically significant (*p* = 0.74).

#### 3.2.4. Standing Long Jump

Figure 3 (with full numerical details in Table 3) shows that SLJ performance increased significantly in the ES + BFR group (+4.0 cm, *p* = 0.03, *d* = 1.25, 95% CI [0.32, 2.17]) but not in ESA (+2.0 cm, *p* = 0.10). The control group exhibited a significant decline (−3.4 cm, *p* = 0.04, *d* = −0.99, 95% CI [−1.83, 0.14]). The between-group difference between ES + BFR and ESA was not statistically significant (*p* = 0.39).

#### 3.2.5. Sit-and-Reach

Improvements in flexibility are detailed in Table 3 and illustrated in Figure 3. Flexibility improved significantly in both ES + BFR (+1.7 cm, *p* = 0.02, *d* = 1.11, 95% CI [0.23, 1.99]) and ESA (+0.9 cm, *p* = 0.03, *d* = 1.01, 95% CI [0.10, 1.92]). The control group showed no significant change (*p* = 0.78). No significant between-group difference was observed (*p* = 0.95).

#### 3.2.6. Body Mass Index

As summarized in Table 3 (and visually confirmed in Figure 3). No significant pre–post changes in BMI were observed in any group (all *p* > 0.19).

No adverse events, including dizziness, excessive pain, numbness, bruising, and skin irritation, were reported during the intervention period. However, given the small sample size, this finding should not be interpreted as definitive evidence of safety.

**Table 3:** Mean and standard deviation (SD) of pre- and post-intervention physical fitness values for three groups.

Variable	Group	Pre		Post		Cohen’s *d* [95% CI]
		Mean ± SD	Mean 95%CI [LL, UL]	Mean ± SD	Mean 95%CI [LL, UL]	
FVC (mL)	ES + BFR	3062.4 ± 559.8	2674.5, 3450.3	3536.9 ± 590.5 ***	3127.7, 3946.1	2.48 [1.08, 3.88]
	ESA	3421.0 ± 242.7	324.1, 3600.7	3674.4 ± 228.9 ***	3504.9, 3844.0	2.68 [1.09, 4.26]
	Control	3153.6 ± 689.4	2676.0, 3631.3	3076.5 ± 649.0	2626.8, 3526.2	—
1000 m Run (s)	ES + BFR	258.5 ± 10.7	250.1, 265.9	252.2 ± 8.3 ***	246.5, 258.0	−2.14 [−3.40, −0.88]
	ESA	258.9 ± 14.0	245.9, 271.8	255.4 ± 12.1 **	244.2, 266.6	−1.66 [−2.80, −0.52]
	Control	256.5 ± 10.3	247.9, 265.1	257.1 ± 9.9	248.8, 265.4	—
50 m Sprint (s)	ES + BFR	7.9 ± 0.2	7.7, 8.0	7.7 ± 0.2 ***	7.6, 7.8	−2.21 [−3.50, −0.92]
	ESA	7.9 ± 0.2	7.7, 8.1	7.7 ± 0.2 **	7.6, 7.9	−1.89 [−3.43, −0.32]
	Control	7.8 ± 0.3	7.6, 8.0	7.8 ± 0.3	7.6, 8.0	—
SLJ (cm)	ES + BFR	225.9 ± 18.8	212.8, 238.9	229.9 ± 18.6 *	217.0, 242.8	1.25 [0.32, 2.17]
	ESA	237.3 ± 12.2	228.3, 246.3	239.3 ± 12.0	230.4, 248.2	—
	Control	230.4 ± 9.5	223.8, 236.9	227.0 ± 11.7 *	218.9, 235.1	−0.99 [−1.83, 0.14]
Sit and Reach (cm)	ES + BFR	8.8 ± 3.7	6.2, 11.3	10.5 ± 3.6 *	7.9, 12	1.11 [0.23, 1.99]
	ESA	11.5 ± 4.1	8.4, 14.5	12.4 ± 4.5 *	9.1, 15.7	1.01 [0.10, 1.92]
	Control	9.8 ± 4.2	6.8, 12.7	9.5 ± 4.7	6.2, 12.7	—
BMI (kg/m^2^)	ES + BFR	22.9 ± 1.8	21.6, 24.2	22.8 ± 1.7	21.6, 24.0	—
	ESA	22.9 ± 1.9	21.4, 24.3	22.8 ± 1.8	21.4, 24.1	—
	Control	23.3 ± 2.4	21.6, 25.0	23.5 ± 2.4	21.8, 25.2	—

Note: FVC = Forced Vital Capacity; SLJ = Standing Long Jump; BMI = Body Mass Index; ES + BFR = stair-climbing exercise snacks with blood flow restriction; ESA = stair-climbing exercise snacks alone; LL = lower limit; UL = upper limit. * *p* < 0.05, ** *p* < 0.01, *** *p* < 0.001 vs. baseline within the same group; ”—” denotes non-significant change. Cohen’s *d* is reported only for significant within-group changes.

## 4. Discussion

This pilot study investigated the effects of a four-week stair-climbing intervention, delivered as a standalone exercise snack (ESA) or in combination with practical blood flow restriction (ES + BFR), on physical fitness in sedentary male university students. Both intervention groups demonstrated significant improvements in FVC, 1000 m run time, 50 m sprint time, and sit-and-reach performance. ES + BFR produced numerically larger within-group effect sizes than ESA for the 1000 m and 50 m sprints, but between-group differences were not statistically significant. Notably, only the ES + BFR group showed a statistically significant improvement in SLJ, whereas the control group exhibited a significant decline on this measure. BMI remained unchanged across all groups. As a pilot trial, these findings are preliminary and warrant further investigation in larger trials.

### 4.1. Pulmonary Function

Both ES + BFR and ESA significantly increased FVC after four weeks, whereas no change was observed in the control group. Repeated short bouts of moderate- to high-intensity exercise likely increase ventilatory demand and respiratory muscle activation [15,16], which could have contributed to the observed FVC changes. FVC is an indirect measure of pulmonary function and does not directly assess cardiorespiratory fitness (e.g., VO_2_max) [34]. Improvements in FVC may reflect enhanced respiratory muscle function rather than central cardiovascular adaptations, and the four-week intervention duration may be insufficient to detect the full extent of cardiopulmonary remodeling. Nevertheless, these exploratory data suggest that stair-climbing exercise snacks may represent a time-efficient strategy to elicit early pulmonary benefits in otherwise inactive students.

### 4.2. Endurance Performance

Both intervention groups improved 1000 m run performance, with ES + BFR showing a larger within-group effect size than ESA. However, because the between-group difference was not statistically significant, this finding should not be interpreted as evidence of superiority for ES + BFR over ESA. Rather, it suggests a possible signal that warrants confirmation in larger trials.

The 1000 m run is a field-based endurance performance test rather than a direct measure of aerobic capacity (e.g., VO_2_max). Consequently, observed improvement may reflect enhanced endurance performance, pacing, neuromuscular efficiency, or familiarity with high-intensity stair-climbing efforts. Given that VO_2_max, cardiac output, muscle oxidative capacity, and blood biomarkers were not assessed, the physiological mechanisms underlying these changes remain speculative.

Previous stair-climbing, high-intensity interval, and BFR studies provide plausible explanations for the observed endurance-performance changes. The maximal stair-climbing protocol may optimize central adaptations. Similar protocols have been shown to rapidly increase VO_2_max and cardiorespiratory fitness within a few weeks despite minimal training volume, indicating rapid central adaptations such as improved oxygen delivery and cardiac function [15]. Furthermore, four weeks of low-load BFR training has been reported to enhance oxygen supply to the exercising musculature, as evidenced by increased oxygenated and total hemoglobin responses at task failure during endurance testing [35]. Additionally, combining stair climbing with BFR may potentiate peripheral adaptations critical for endurance. Repeated maximal stair-climbing efforts impose high metabolic stress on leg muscles, driving improvements in skeletal-muscle oxidative capacity. As demonstrated in classic sprint-interval studies, even as few as six sessions over ~2 weeks can enhance the ability to sustain high workloads without premature fatigue [36].

Collectively, these findings align with the possibility that stair-climbing exercise snacks with or without BFR can yield early improvements in endurance performance. Future fully powered trials should incorporate direct physiological assessments to determine whether changes in VO_2_max, oxygen delivery, or muscle oxidative function contribute to these performance outcomes.

### 4.3. Sprint Performance and Lower-Limb Power

Both intervention groups improved 50 m sprint performance, with ES + BFR showing a larger within-group effect than ESA; however, the between-group difference was not statistically significant. Only the ES + BFR group demonstrated a significant improvement in SLJ, whereas the ESA group remained at the baseline and the control group declined.

An interpretation is that repeated maximal-effort stair climbing provided a task-specific stimulus involving rapid force production and repeated lower-limb propulsion. Prior work in inactive young adults indicates that similar accumulated stair-climbing protocols can elevate peak power within a few weeks, indicating that short bouts are sufficient to induce neuromuscular adaptations [15] such as with respect to neuromuscular coordination and explosive power [37]. Furthermore, BFR is widely reported to enhance fast-twitch fiber recruitment and sprint-related performance [38]. Our findings imply that adding BFR to stair-climbing exercise snacks may further amplify explosive and speed-related adaptations. This aligns with the broader literature indicating that BFR can markedly improve sprint performance, muscle strength, and hypertrophy [20,39]. Nonetheless, longer interventions with direct measures of strength, muscle morphology, and neuromuscular activation are required to clarify the mechanisms.

Although the ESA group did not exhibit SLJ gains, it did not experience the decline observed in the control group, suggesting that four weeks of maximal-effort stair-climbing exercise snacks may help preserve lower-limb power in sedentary students.

### 4.4. Flexibility

Both intervention groups showed clear improvement in sit-and-reach performance after four weeks, whereas the control group did not. The sit-and-reach test serves as a field measure of posterior-chain flexibility, particularly hamstring and lumbosacral flexibility, with moderate criterion-related validity for estimating hamstring extensibility in young adults [30].

Sedentary students often present reduced hamstring flexibility and restricted hip/trunk mobility, with prolonged sitting linked to increased posterior-chain stiffness [40]. The flexibility gains observed in the intervention groups may indicate that the stair-climbing intervention elicited a sufficient stimulus to counteract posterior-chain stiffness related to sedentariness. Short-term gains in flexibility may primarily reflect enhanced stretch tolerance rather than structural lengthening alone [41]. Because stretch tolerance was not directly measured, this interpretation should be regarded as speculative. Nevertheless, improved flexibility may facilitate daily movements and reduce perceived musculoskeletal discomfort.

### 4.5. Body Mass Index

No significant changes in BMI were observed in any group, reinforcing the interpretation that the primary benefits of the intervention groups were functional (cardiorespiratory and neuromuscular) rather than anthropometric in the short term. This finding is consistent with previous research. A similar short-term study using exercise snacks with BFR in inactive students also reported no meaningful changes in body composition [42], which is expected, given that detectable alterations typically require interventions lasting at least 8~12 weeks [10]. Meta-analyses further indicate that short-term accumulated exercise produces only minimal or non-significant changes in body weight, BMI, and fat mass [11].

### 4.6. Limitations

Several limitations of this pilot study should be considered. The per-protocol sample (*n* = 23) fell short of the planned size, which limited statistical power and likely produced inflated effect-size estimates, as indicated by wide 95% confidence intervals [33]. The sample was restricted to male university students, reducing generalizability, and the demanding 48-session supervised protocol may have introduced selection bias by favoring highly motivated participants. The blood flow restriction stimulus was not individually standardized; reliance on perceived tightness (~7/10) rather than actual occlusion pressure [23] probably introduced substantial interindividual variability in the elicited physiological adaptation. The trial was not prospectively registered, reducing transparency and increasing the risk of reporting bias, though the study was exploratory in nature. From a measurement perspective, the absence of direct physiological outcomes such as VO_2_max, muscle biopsy, electromyography, or blood biomarkers renders mechanistic interpretations necessarily speculative. Forced vital capacity is an indirect measure of pulmonary function and cannot substitute for a direct assessment of cardiorespiratory fitness, while the 1000 m run, although validated in Chinese university students, lacks extensive calibration against laboratory gold standards. Dietary intake was captured solely through weekly self-report logs, without objective verification. To address these limitations, future work should recruit larger, more diverse cohorts that include women, employ interventions of at least eight weeks with follow-up assessments, individualize blood flow restriction pressure using Doppler ultrasound-derived limb occlusion pressure, ensure prospective registration, and incorporate direct physiological measurements to clarify the underlying mechanisms.

## 5. Conclusions

This pilot study suggests that a four-week maximal-effort stair-climbing exercise-snack protocol is feasible and may improve pulmonary function, endurance, speed, power, and flexibility in sedentary male university students. Adding practical BFR yielded promising numerical effects for speed, power, and endurance outcomes but without significant between-group differences. These findings should be interpreted as preliminary and hypothesis-generating. Larger, prospectively registered trails with objectively standardized BFR pressure, direct physiological measurements, and more diverse samples are needed to determine whether BFR provides meaningful benefits beyond exercise snacks alone.

## Figures and Tables

**Table 1:** Baseline characteristics of participants by group. Values are presented as mean ± standard deviation.

Variable	ES + BFR (*n* = 8)	ESA (*n* = 7)	Control (*n* = 8)	*p*
Age (year)	21.00 ± 0.76	20.86 ± 0.38	21.75 ± 1.28	0.15
Height (cm)	173.93 ± 2.96	177.96 ± 2.56	174.83 ± 6.26	0.67
Weight (kg)	69.26 ± 5.26	72.64 ± 7.49	71.33 ± 9.61	0.91
Supervised sessions attended	47.63 ± 0.74	47.29 ± 1.25	N/A	0.45

Note: ES + BFR = stair-climbing exercise snacks with blood flow restriction; ESA = stair-climbing exercise snacks alone.

**Table 2:** ANOVA outcomes of physical fitness values for three groups.

Variable	Group F, *p*	Time F, *p*	G × T F, *p*	η^2^p (G × T)
FVC (mL)	F = 1.24, *p* = 0.31	F = 41.53, *p* < 0.001	F = 23.82, *p* < 0.001	0.70
1000 m run (s)	F = 0.06, *p* = 0.94	F = 36.66, *p* < 0.001	F = 17.91, *p* < 0.001	0.64
50 m Sprint (s)	F = 0.02, *p* = 0.98	F = 35.86, *p* < 0.001	F = 16.12, *p* < 0.001	0.62
SLJ (cm)	F = 1.20, *p* = 0.32	F = 1.69, *p* = 0.21	F = 11.13, *p* = 0.001	0.53
Sit-and-Reach (cm)	F = 0.78, *p* = 0.47	F = 8.92, *p* = 0.007	F = 5.42, *p* = 0.01	0.35
BMI (kg/m^2^)	F = 0.21, *p* = 0.82	F = 0.10, *p* = 0.75	F = 2.28, *p* = 0.13	—

Note: BMI = Body Mass Index; FVC = Forced Vital Capacity; SLJ = Standing Long Jump; ES + BFR = stair-climbing exercise snacks with blood flow restriction; ESA = stair-climbing exercise snacks alone; ANOVA = analysis of variance; G × T = Group × Time; η^2^p = partial eta squared; “—” denotes non-significant change.

## Data Availability

The datasets generated and/or analyzed during the current study are not publicly available due to participant privacy and ethical restrictions but are available from the corresponding author upon reasonable request.
